# A Bibliometric and Visualized Analysis of Uremic Cardiomyopathy From 1990 to 2021

**DOI:** 10.3389/fcvm.2022.908040

**Published:** 2022-07-12

**Authors:** Jing-Fu Bao, Pan-Pan Hu, Qin-Ying She, Difei Zhang, Jia-Ju Mo, Aiqing Li

**Affiliations:** ^1^State Key Laboratory of Organ Failure Research, National Clinical Research Center for Kidney Disease, Nanfang Hospital, Southern Medical University, Guangzhou, China; ^2^Guangdong Provincial Key Laboratory of Renal Failure Research, Guangzhou Regenerative Medicine and Health Guangdong Laboratory, Guangzhou, China; ^3^Department of Nephrology, The Fifth Affiliated Hospital, Southern Medical University, Guangzhou, China; ^4^Department of Nephrology, Guangdong Provincial Hospital of Chinese Medicine, Guangzhou, China

**Keywords:** bibliometric analysis, uremic cardiomyopathy, CiteSpace, VOSviewer, HistCite, bibliometrix

## Abstract

**Background:**

Uremic cardiomyopathy is commonly presented in chronic kidney disease (CKD), and it severely affects the prognosis of patients with CKD. In the past few decades, the investigation of uremic cardiomyopathy has developed rapidly. However, no report has summarized the situation of uremic cardiomyopathy research to date. This study aimed to evaluate the state of uremic cardiomyopathy research in the last 30 years and identify important topics and achievements, as well as emerging trends through bibliometric analysis.

**Materials and Methods:**

Publications related to uremic cardiomyopathy were collected from Science Citation Index Expanded. HistCite, VOSviewer, CiteSpace, and the Bibliometrix Package were used for bibliometric analysis and visualization, including the analysis of the overall distribution of the annual publication, leading countries, and active institutions and authors, core journals, co-cited references, and keywords.

**Results:**

A total of 2,403 studies related to uremic cardiomyopathy were obtained, and progress related to uremic cardiomyopathy was slower in past 3 years. A total of 10,077 authors from 2,697 institutions in 89 countries or regions reported investigations on uremic cardiomyopathy. The United States of America was the most productive and the most cited country. Myles Wolf, Joseph I Shapiro, and Carmine Zoccali published most articles in uremic cardiomyopathy, and journals in nephrology possessed core status in the field. Phosphate metabolism was the hotspot in uremic cardiomyopathy research in recent years, and future progress may concentrate on phosphate metabolism, endogenous natriuretic factors, and novel biomarkers.

**Conclusion:**

The United States of America and European countries played central roles in uremic cardiomyopathy research, while Chinese scholars should be more involved in this field. Global publications on uremic cardiomyopathy have entered platform stage, and the fibroblast growth factor-23-klotho axis remained a hotspot in this field. Endogenous natriuretic factors and novel biomarkers may be potential directions in future investigations.

## Introduction

Cardiovascular disease is a leading cause of death in patients with chronic kidney disease (CKD) (1). The most common feature of cardiovascular changes in CKD is uremic cardiomyopathy (2), which presents even in the early stages of CKD and in up to 75% of patients with predialysis (3–5). Uremic cardiomyopathy contributes to more severe cardiovascular anomalies, including diastolic dysfunction, arrhythmia, and even sudden death (6). Hence, uremic cardiomyopathy has been widely concerned and is considered as a main therapeutic target in patients with CKD with cardiovascular complications.

Several factors are highly involved in the emergence of uremic cardiomyopathy, including hypertension, anemia, renin-angiotensin system overactivation, microinflammation, CKD mineral-bone disorder (CKD-MBD), and uremic toxins (7). Importantly, these factors constitute the basis of uremic cardiomyopathy treatment. Since Parfrey et al. (8) observed the regression of LVH after renal transplantation, preventing uremic cardiomyopathy has become a possibility. Subsequently, recombinant human erythropoietin (9), hemodialysis (10), and spironolactone (11) were all found to cause uremic cardiomyopathy regression in patients with CKD. In 2011, Faul et al. (12) identified that fibroblast growth factor 23 (FGF-23), which is elevated in CKD, can directly induce LVH. This further links CKD-MBD to uremic cardiomyopathy and implies another therapeutic strategy for uremic cardiomyopathy. However, the lack of full understanding of the pathophysiological process of uremic cardiomyopathy makes the related progress slower. For example, sustained erythropoietin treatment may bring side effects (e.g., hypertension and thrombosis) and increase mortality (13), and FGF-23 neutralization failed to mitigate LVH but increased aortic calcification and the risk of mortality in CKD rats (14). Hence, comprehension of the developmental and potential directions of uremic cardiomyopathy to deepen our understanding and propose novel therapeutic strategies in this field are required.

Bibliometric analysis is “*the application of mathematical and statistical methods to books and other media of communication*” (15), and it can be used as an analytic model for quantitatively analyzing scientific publications (16), which are medium of scientific information. As an analytic model, bibliometric analysis can quickly reveal the knowledge network, research topic evolvement, and potential research directions in a certain research field (17). Liu et al. (18) analyzed podocyte injury research through bibliometric analysis and identified the most prolific country, top contributing authors, and most popular journals in this field. Most importantly, they also provided research topic evolvement, namely, “diabetic nephropathy” to “autophagy, microRNA, and inflammation,” and identified that traditional Chinese medicine may be a potential direction in future podocyte injury research. Hence, bibliometrics can act as a critical approach to provide an in-depth evaluation of the development of the uremic cardiomyopathy field, which may guide us in future work. To our knowledge, no bibliometric analysis of uremic cardiomyopathy has been performed. We aim to analyze publications on uremic cardiomyopathy and evaluate the status and emerging trends in this field.

## Materials and Methods

### Search Strategy in Web of Science

Although PubMed contains more biomedicine-related publications compared to web of science (WoS), the latter has a more complete citation network than PubMed, and is critical for co-citation analysis. WoS almost includes the most influential publications in uremic cardiomyopathy, which avoids the omission of important studies as far as possible. In addition, HistCite, VOSviewer, CiteSpace, and Bibliometrix packages are most suitable for analyzing datasets derived from WoS. Based on these, we chose a dataset derived from WoS as our analytic target. WoS’ topic search is equivalent to a search model of words in the title, abstracts, author keywords, and keywords plus, thus, we chose topic search to precisely obtain the topic. As LVH is the main feature of uremic cardiomyopathy (7), we also searched “[(chronic kidney disease) OR (chronic renal failure)] AND [(cardiac hypertrophy) OR (left ventricular hypertrophy)]” in addition to “(uremic cardiomyopathy) OR (uremic cardiac hypertrophy).” Importantly, CKD and LVH can be jointly observed in Fabry disease, which is a lysosomal storage disease that affects both the kidney and the heart (19). Hence, we excluded Fabry disease in our dataset.

We performed a literature search on the Science Citation Index Expanded (SCIE), and the search formula was as follows: “((((chronic NEAR/0 kidney NEAR/0 disease) OR (chronic NEAR/0 renal NEAR/0 failure)) AND ((cardiac NEAR/0 hypertrophy) OR (left NEAR/0 ventricular NEAR/0 hypertrophy)) OR ((uremic NEAR/0 cardiac NEAR/0 hypertrophy) OR (uremic NEAR/0 cardiomyopathy))) NOT (Fabry))”. The publication year was restricted to 1990–2021, and the document types were “articles” or “reviews”. In addition, the article language was restricted to English. All the information, including publication number and related titles, authors, affiliations, countries, keywords, journals, publication year, references, and citations, were collected for analysis. We finally got 2,403 publications. To avoid deviations from updates, all the above operations were completed within 1 day, and on May 22, 2022.

### Bibliometric Analysis

HistCite (version 12.03.17) (20) was used to analyze the publication number, total global citation score (TGCS), and total local citation score (TLCS) for each publication year, active countries, active institutions, active authors, and core journals. TGCS is the number of citations in the SCIE, while TLCS is the number of citations in the current publication set.

Co-citation and co-occurrence networks were completed by VOSviewer (version 1.6.18) (21). Collaboration between countries, institutions, and authors and mutual citation between journals were analyzed by co-citation networks. Keywords occurrence in various publications was analyzed by co-occurrence networks. The size of the nodes in networks represents publication number, and the thickness of the connecting line in networks means the strength of the node-link. The threshold (the minimum number of documents and the maximum number of total link strength) of co-citation and co-occurrence networks were presented in corresponding parts. The size of the circle of an item was proportional to its number of publications, and the width of the line between the two items was proportional to the magnitude of linkages. In addition, the same color indicates close cooperation, and the total link strength of an item reflects the degree of cooperation with others.

CiteSpace (version 5.8.R3) (22) was used to analyze the knowledge domain in uremic cardiomyopathy, including cluster, timeline view, and reference burst analysis. Time slicing was set as from 1990 January to 2021 December, and 2 years per slice. Node type was set as References, and selection criteria were set as top 50 levels of most cited or occurred items from each slice. Modularity Q and mean silhouette were used to evaluate the reliability of clustering; Q > 0.3 and mean silhouette of >0.5 indicate ample clustering structure and convincing clustering results, respectively.

Author’s production over time and the thematic evolution analysis were performed by R language-based the Bibliometrix Package (4.1.0 Package) (23), which can show the publications of most 10 productive authors for the last 20 years and identify research topic evolvement, respectively.

## Results

### The Overview of Publications on Uremic Cardiomyopathy

**FIGURE 1:**
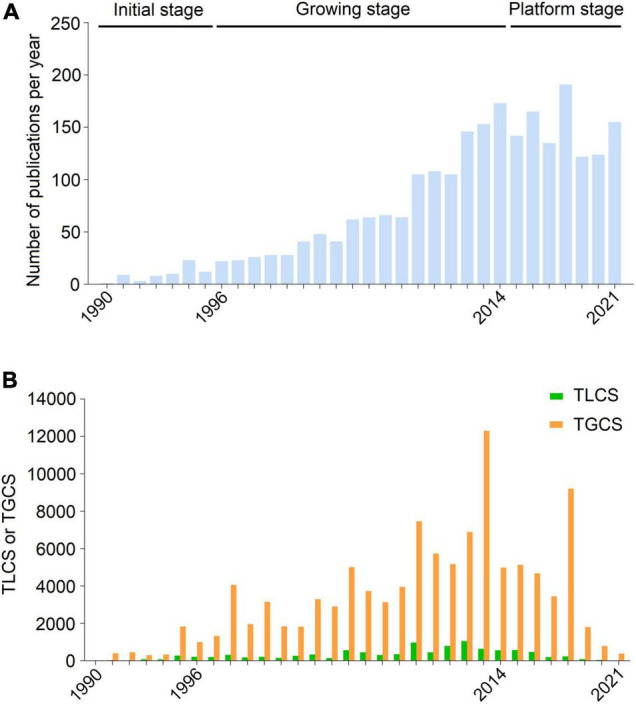
Publication outputs and citations on uremic cardiomyopathy. **(A)** Global annual production trends. **(B)** Annual total global citation score (TGCS) and total local citation score (TLCS) of publications on uremic cardiomyopathy.

A total of 2,403 studies related to uremic cardiomyopathy were obtained from SCIE, including 1,810 articles and 593 reviews (Supplementary Table 1). From only one publication in 1990 to 155 publications in 2021, global publications in the field demonstrated an overt growth trend (Figure 1A), and this can be divided into three stages according to the annual production status: Initial stage (1990–1996), and growing stage (1997–2014), and platform stage (2015–2021). The number of publications was less than 30 per year in the initial stage and a significant increase in publication number with an average growth rate of 12.90% per year in the growing stage. However, the average growth rate of publications on uremic cardiomyopathy dropped to 1.47% during the period from 2015 to 2021 (Figure 1A).

Publications on uremic cardiomyopathy have been cited 90,649 times on SCIE, with an average of 45.16 times per article. Corresponding to publication number, the TGCS of publications was very low in the initial stage, while TGCS exhibited a gradually increasing trend from 1997 to 2014 and peaked in 2013. Since 2015, the TGCS was relatively stable (Figure 1B), further indicating the platform stage in this period.

### Leading Countries in the Uremic Cardiomyopathy Field

**FIGURE 2:**
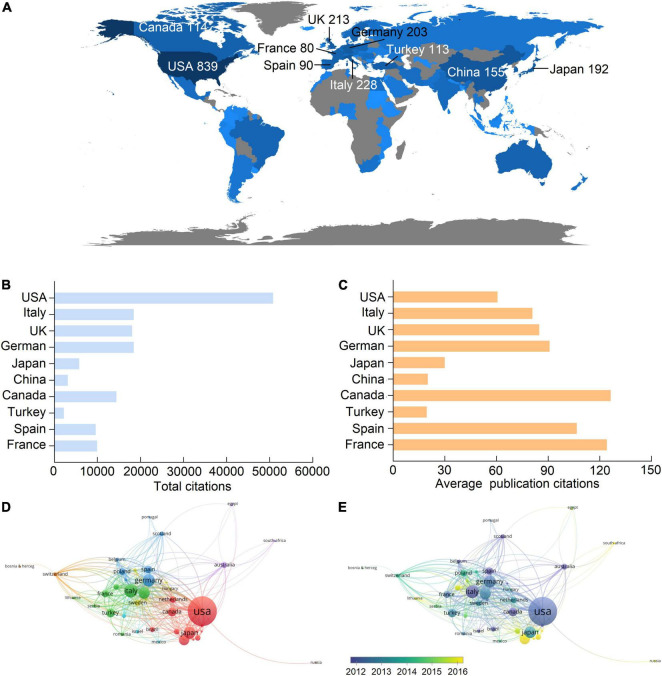
Leading countries in the uremic cardiomyopathy field. **(A)** Worldwide distribution of the publication. **(B,C)** Total citations and average publication citations of the top 10 productive countries. **(D)** Clustering of collaboration among countries. **(E)** Timeline visualization of collaboration among countries.

A total of 89 countries and regions contributed to publications in this field, and the United States of America (USA) contributed the largest number of publications (839, 34.9% of all articles), followed by Italy (228, 9.5%), United Kingdom (UK) (213, 8.9%), Germany (203, 8.4%), and Japan (192, 8%) (Figure 2A and Table 1). Correspondingly, studies from the United States had the highest number of citations (50,802 citations), followed by those from Germany (18,429 citations), Italy (18,420 citations), the United Kingdom (18,057 citations), and Canada (14,399 citations), and the rest are all less than 10,000 citations (Figure 2B). Notably, although Japan, China, and Turkey had higher publication numbers, their total citations and average citations were significantly lower than others in the top 10 productive countries (Figures 2B,C), indicating a minor influence on their publications.

**TABLE 1:** The top 10 productive countries concerning uremic cardiomyopathy.

Rank	Country	Publications (*n* %)	TLCS	TGCS	Average citation
1	United States	839 (34.9)	5,309	50,802	60.6
2	Italy	228 (9.5)	1,163	18,420	80.8
3	United Kingdom	213 (8.9)	1,179	18,057	84.8
4	Germany	203 (8.5)	1,587	18,429	90.8
5	Japan	192 (8.0)	546	5,747	29.9
6	China	155 (6.5)	189	3,112	20.1
7	Canada	114 (4.7)	1,341	14,399	126.3
8	Turkey	113 (4.7)	234	2,200	19.5
9	Spain	90 (3.7)	387	9,595	106.6
10	France	80 (3.3)	436	9,923	124.0

There were 44 countries with more than five publications that have been included in the co-authorship analysis, and one of them was not connected to others. Hence, 43 countries were used to analyze collaboration among countries. The highest total link strength was the United States (total link strength = 390 times), indicating the largest cooperative network led by it (Figure 2D). In this cooperative network, Germany (49), Canada (41), Italy (37), and China (33) had the closest cooperation with the United States (Figure 2D). Among the top 10 productive countries (Figure 2A and Table 1), China is the late starter in international cooperation in this field (Figure 2E).

### Active Institutes and Authors in the Uremic Cardiomyopathy Field

There were 10,077 authors from 2,697 institutions who have published articles on uremic cardiomyopathy. The top 10 productive institutions were almost American universities, except Karolinska Institute. The University of Miami was the leading institution in publication, followed by the University of Pennsylvania, the University of Washington, Harvard University, and Northwestern University (Table 2). Both the TGCS and TLCS of the University of Miami (cited 6,022 times) were the highest in the top 10 active institutes (Table 2), indicating their leading role in uremic cardiomyopathy.

**TABLE 2:** The top 10 productive institutions concerning uremic cardiomyopathy.

Rank	Institution	Publications	TLCS	TGCS	Average citation
1	University of Miami	54	1,496	6,032	111.7
2	University of Pennsylvania	45	809	3,547	78.8
3	University of Washington	45	427	4,068	90.4
4	Harvard University	44	929	4,602	104.6
5	Northwestern University	42	471	2,157	51.4
6	University of Alabama Birmingham	38	481	4,644	122.2
7	Duke University	37	144	1,676	45.3
8	University of California, San Francisco	37	349	2,024	54.7
9	Johns Hopkins University	32	274	4,444	138.9
10	Karolinska Institution	32	128	1,317	41.2

**FIGURE 3:**
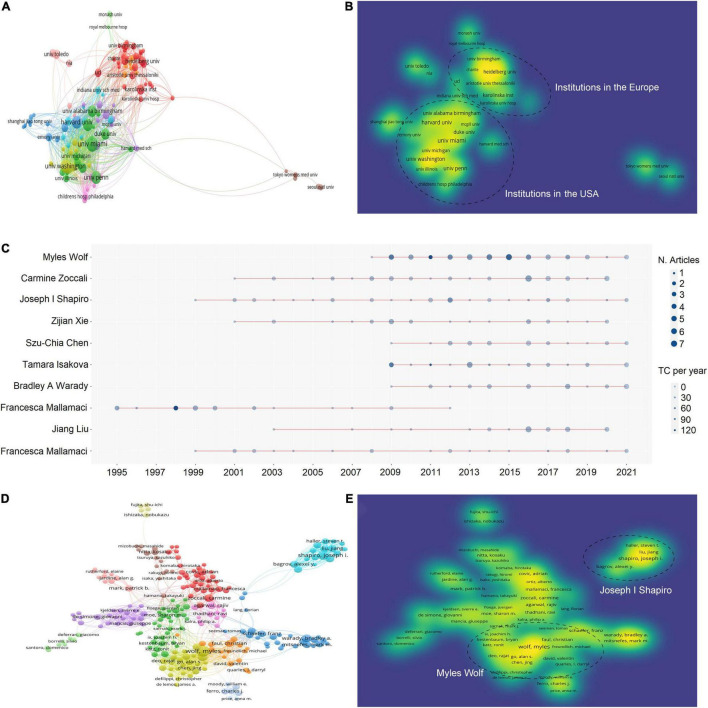
Active institutes and authors analysis. **(A)** Clustering of collaboration among institutes. **(B)** Density visualization of institutes based on the mean frequency of appearance and two groups with higher collaboration can be observed. **(C)** Timeline distribution of publications from the top 10 active authors. **(D)** Clustering of collaboration among authors. **(E)** Density visualization of authors based on the mean frequency of appearance and three groups with higher collaboration can be observed.

We analyzed the co-authorship of 121 institutions with more than 10 publications, while the items that were not connected were removed, leaving a total of 119 institutions. The University of Pennsylvania showed the most frequent collaboration with other institutions (total link strength = 186 times) (Figure 3A), which was followed by the University of Washington (162), the University of California, San Francisco (138), the University of Illinois (112), and Tulane University (101). Notably, the collaboration was mostly taking place within the institutions in the United States or the institutions in European (Figure 3B).

The top 10 active authors were Myles Wolf of the University of Miami, Joseph I Shapiro of Marshall University, Carmine Zoccali of National Research Council, Zijian Xie of Marshall University, Szu-Chia Chen of Kaohsiung Medical University, Tamara Isakova of Northwestern University, Bradley A. Warady of Children’s Mercy Hospital, Robert N. Foley of USRDS Coordinating Center, Jiang Liu of Marshall University, and Francesca Mallamaci of National Research Council of Italy (Table 3). Of the top 10 active authors, Carmine Zoccali and Francesca Mallamaci had the longest period in publication on uremic cardiomyopathy (Figure 3C), and they both focused mainly on clinical features of uremic cardiomyopathy.

**TABLE 3:** The top 10 active authors concerning uremic cardiomyopathy.

Rank	Name	Publications	TLCS	TGCS	H index
1	Myles Wolf	46	1,439	5,749	33
2	Joseph I. Shapiro	40	337	1,550	23
3	Carmine Zoccali	33	243	1,973	21
4	Zijian Xie	25	265	1,250	19
5	Szu-Chia Chen	23	77	427	11
6	Tamara Isakova	23	988	3,473	17
7	Bradley A. Warady	23	100	681	17
8	Robert N. Foley	22	761	5,456	15
9	Jiang Liu	22	112	621	14
10	Francesca Mallamaci	22	88	878	14

Moreover, a total of 304 authors were co-authored in more than five publications, excluding 72 authors that were not connected. The two authors with the highest total link strength constructed the biggest two collaboration networks, namely, Myles Wolf (total link strength = 186 times) and Joseph I. Shapiro (138) (Figure 3D). The collaboration network of Myles Wolf is mainly concentrated on the relationship between phosphate metabolism and uremic cardiomyopathy, while the collaboration network of Joseph I. Shapiro is mainly concentrated on the relationship between Na^+^/K^+^-ATPase and uremic cardiomyopathy. These collaboration networks represent two main topics in uremic cardiomyopathy. Interestingly, the collaboration network of Joseph I. Shapiro demonstrated lower cooperation with other networks (Figure 3E).

### Core Journals in the Uremic Cardiomyopathy Field

Publications on uremic cardiomyopathy were presented in 500 journals. The top 10 journals with the highest publications were shown in Table 4, and about 28.3% of all publications were published in these journals. In addition, about 42% of all citations occurred in the top 10 journals with the highest publications, indicating their higher influence in the uremic cardiomyopathy field. It is noteworthy that most of these 10 journals are nephrology-related, except the Journal of Hypertension and PLoS ONE. American Journal of Kidney Diseases had the largest number of citations (8,559 citations), followed by the Journal of the American Society of Nephrology (8,447), Kidney International (7,854), Journal of Hypertension (6,576), and Nephrology Dialysis Transplantation (6,209).

**TABLE 4:** The top 10 journals concerning uremic cardiomyopathy.

Rank	Journal	Counts	Impact factor (2020)	TLCS	TGCS	H index
1	Nephrology Dialysis Transplantation	131	5.992	826	6209	44
2	Kidney International	93	10.612	1068	7854	50
3	Pediatric Nephrology	84	3.714	302	1870	24
4	American Journal of Kidney Diseases	79	8.860	937	8559	43
5	Journal of the American Society of Nephrology	62	10.121	1249	8447	42
6	Journal of Hypertension	57	4.844	272	6576	24
7	PLoS ONE	50	3.240	0	1476	12
8	Clinical Journal of the American Society of Nephrology	46	8.237	494	2452	30
9	Current Opinion in Nephrology and Hypertension	40	2.894	145	1154	22
10	Seminars in Dialysis	39	3.455	107	1026	16

**FIGURE 4:**
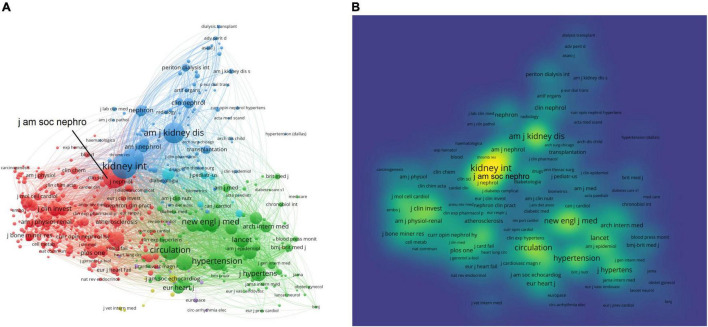
Core journals in the uremic cardiomyopathy field. **(A)** Clustering of co-citation among journals. **(B)** Density visualization of journals based on the mean frequency of appearance, and journals in nephrology possessed a core position in clustering.

A total of 553 journals were co-cited in more than 20 publications. Kidney International (total link strength = 1,080,090 times), Hypertension (893,963), Journal of the American Society of Nephrology (819,058), Circulation (788,881), and New England Journal of Medicine (742,818) showed the most co-citations with other journals (Figure 4A). Interestingly, journals in the nephrology field demonstrated a leading role in the co-citation network as indicated by the most complex citation network (Figure 4A) and the highest density in the co-citation network (Kidney International, Journal of the American Society of Nephrology, and American Journal of Kidney Diseases) (Figure 4B).

### Co-cited Publications in the Uremic Cardiomyopathy Field

The top 10 most cited publications were listed in Supplementary Table 2. Among these publications, there were two research articles, five guidelines, and three reviews, and seven publications were less cited by uremic cardiomyopathy-related publications (TLCS < 50), implying an indirect relationship with uremic cardiomyopathy. Thus, we further analyzed the top 10 most TLCS publications (Table 5). There were eight research articles and two review, and most of them were epidemiological reports, while three publications were pathophysiological investigations. Interestingly, these pathophysiological investigations all concentrated on the relationship between FGF-23 and uremic cardiomyopathy (12, 14, 24).

**TABLE 5:** The top 10 publications with the highest total local citation score (TLCS).

Rank	References	Journal	Year	Category	TLCS	TGCS
1	Faul et al. (12)	Journal of Clinical Investigation	2011	Epidemiology/ Pathophysiology	373	1,320
2	Gutiérrez et al. (27)	Circulation	2009	Epidemiology	271	621
3	Foley (52)	American Journal of Kidney Diseases	1998	Review-Epidemiology	190	2,631
4	Foley (2)	Journal of the American Society of Nephrology	1995	Epidemiology	148	422
5	Grabner et al. (24)	Cell Metabolism	2015	Pathophysiology	121	311
6	Parfrey (53)	Nephrology Dialysis Transplantation	1996	Epidemiology	119	498
7	Glassock (54)	Clinical Journal of the American Society of Nephrology	2009	Review-Epidemiology	91	238
8	Shalhoub et al. (14)	Journal of Clinical Investigation	2012	Pathophysiology	90	280
9	Paoletti et al. (32)	American Journal of Kidney Diseases	2005	Epidemiology	85	149
10	Thadhani et al. (43)	Journal of the American Medical Association	2012	Randomized Controlled Trial	81	388

**FIGURE 5:**
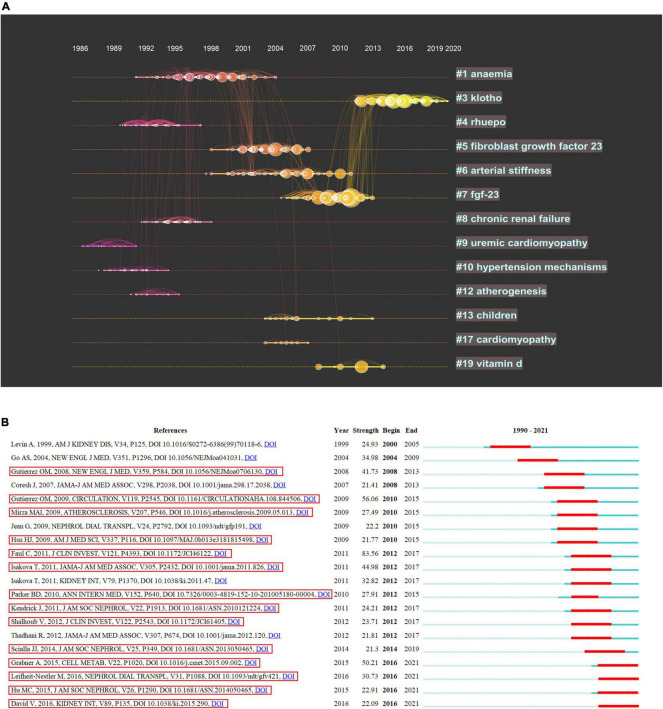
Co-cited reference analysis. **(A)** Timeline distribution of 13 clusters with the higher K values. **(B)** The top 20 references with the higher citation bursts, references with the red box is directly associated with FGF-23.

The visualization network of cited references demonstrated 13 clusters (Table 6), the modularity Q was 0.8555, and the mean silhouette value was 0.9436. We performed a visualized timeline for these clusters to identify the involvement of the research topic in the uremic cardiomyopathy field. “Anemia” and “rhuepo (recombinant human erythropoietin)” were early fields in uremic cardiomyopathy (Cluster ID #1 and ID #4, Figure 5A), which are attributed to the discovery of anemia-induced cardiac injury in CKD (25) and erythropoietin can mitigate LVH in patients with CKD (9). Later, CKD-MBD was considered a cardiovascular risk factor, and vitamin D and FGF-23 have gained wide concern (26) (Cluster ID #5, ID #7, and ID #19, Figure 5A), especially FGF-23 was found to correlate to LVH and can directly induce LVH (12, 27). In recent years, the hotspot of uremic cardiomyopathy was on “klotho” (Cluster ID #3, Figure 5A), which declines CKD and has FGF-23-dependent and FGF-23-independent protective effects on the myocardium (28).

**TABLE 6:** The 13 clusters of co-cited references with the higher K value.

Cluster ID	Size	Silhouette	Mean year	Top term	Log (likelihood ratio, *p* level)
#1	94	0.908	1997	Anemia	28.05, 1.0E-4
#3	83	0.938	2015	Klotho	27.77, 1.0E-4
#4	69	0.979	1992	Rhuepo	14.47, 0.001
#5	68	0.957	2003	Fibroblast growth factor 23	14.01, 0.001
#6	64	0.923	2003	Arterial stiffness	14.72, 0.001
#7	54	0.958	2009	Fgf-23	25.69, 1.0E-4
#8	51	0.931	1994	Chronic renal failure	13.12, 0.001
#9	43	1	1988	Uremic cardiomyopathy	12.05, 0.001
#10	39	0.955	1990	Hypertension mechanisms	8.84, 0.005
#12	14	1	1992	Atherogenesis	NaN, 0.001
#13	14	0.998	2006	Children	41.94, 1.0E-4
#17	7	1	2005	Cardiomyopathy	8.88, 0.005
#19	6	1	2011	Vitamin d	13.80, 0.001

The term “burst” refers to references that frequently appear over some time and can reflect the hot topics in a certain period based on the topic of references. Reference burst detection of the top 20 references, with the strongest citation bursts, demonstrated that the publication of Faul et al. (12), Gutiérrez et al. (27), and Grabner et al. (24) are the top three burst strength (Figure 5B). Interestingly, these publications were all concentrated on the relationship between FGF-23 and uremic cardiomyopathy, and 14 references were directly correlated with FGF-23 or klotho, suggesting the specific role of calcium-phosphorus metabolism on uremic cardiomyopathy. The recent publications with higher citation bursts were coming from Hu et al. (29), Leifheit-Nestler et al. (30), and David et al. (31); Hu et al. and Leifheit-Nestler et al. demonstrated that LVH in CKD may be attributed to a deficiency of soluble klotho, thus, implying a potential therapeutic strategy in uremic cardiomyopathy. David et al. reported that inflammation and iron deficiency can upregulate FGF-23, which further indicates the association between inflammation, anemia, FGF-23, and cardiac injuries in patients with CKD.

### Keywords of References in the Uremic Cardiomyopathy Field

**FIGURE 6:**
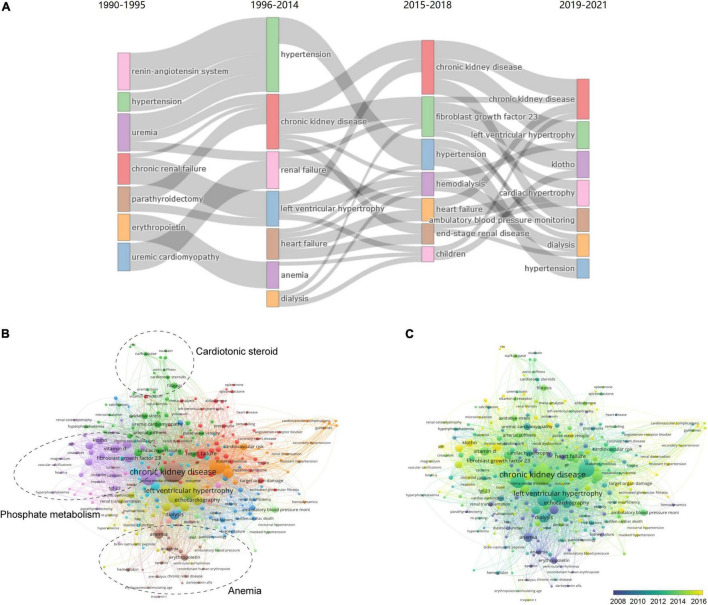
Analysis of keywords in publications and milestones of the uremic cardiomyopathy field. **(A)** Major keywords evolution of uremic cardiomyopathy research. **(B)** Clustering of co-occurrence among keywords. **(C)** Timeline visualization of co-occurrence among keywords.

Thematic evolution analysis of the author’s keywords showed that the initial stage of investigation on uremic cardiomyopathy was mainly focused on “renin-angiotensin system,” “hypertension,” “parathyroidectomy,” and “erythropoietin,” whereas, the main topics remained “hypertension” and “anemia” in the growing stage, indicating that the understanding on uremic cardiomyopathy was not deepened in this stage. In the platform stage, the main topic of uremic cardiomyopathy has gradually evolved toward “fibroblast growth factor-23,” which is a breakthrough in the uremic cardiomyopathy field (12, 27). In the past 3 years, “klotho” attracted the attention of scholars in uremic cardiomyopathy (Figure 6A). These results were consistent with the previous analysis (Figure 5A).

A total of 283 keywords (set as author’s keywords) were identified as having occurred more than five times. It can be found that FGF-23, klotho, and vitamin D, which are all related to phosphate metabolism, constitute a critical part of uremic cardiomyopathy research (Figure 6B), and these were a relatively novel topic in this field as well (Figure 6C). In addition, cardiotonic steroids and Na^+^-K^+^-ATPase constitute another important part of uremic cardiomyopathy research (Figure 6B). Anemia, as a classical topic in uremic cardiomyopathy (Figure 6C), also occupied its domain (Figure 6B). Thus, these three parts were the leading topic in uremic cardiomyopathy.

Notably, we identified several potential novel aspects in uremic cardiomyopathy (Figure 6C), including Na^+^-K^+^-ATPase (occurrences: 16), reactive oxygen species (ROS) (5), troponin T (5), ambulatory blood pressure (10), and nocturnal hypertension (11), which were all less exposed in uremic cardiomyopathy but may have potential significance.

## Discussion

In this study, we analyzed the main knowledge domain and emerging trends of uremic cardiomyopathy through bibliometric analysis. The results showed that annual publications on uremic cardiomyopathy have entered a platform stage since its rise in 1996. CKD-related phosphate metabolism disorder remains the hotspot of uremic cardiomyopathy research, and the klotho is an emerging trend in recent years.

### Leading Countries in Uremic Cardiomyopathy Research

The United States was the most productive country (Figure 2A and Table 1); nine of the top 10 productive institutions and seven of the top 10 productive authors were from the United States (Tables 2, 3). The main research topic in uremic cardiomyopathy, the phosphate metabolism, was almost established by scholars from the United States (Figure 5B). Thus, the United States demonstrates a great impact on uremic cardiomyopathy research. In addition to the United States, scholars in Europe, as represented by Carmine Zoccali, possessed another critical part in uremic cardiomyopathy (Figures 3A,B). Compared to American scholars, European scholars mainly concentered on the clinical features and diagnosis of uremic cardiomyopathy (32, 33). Recently, Lv et al. (34) analyzed publications on cardiorenal syndrome and found that the United States and Europe still play central roles in this field. Although uremic cardiomyopathy only constitutes a small part of a cardiorenal syndrome, our result still partially confirmed their analysis.

Although China has the 6th rank in publications worldwide (Figure 2A and Table 1), the total and average publication citations are relatively lower in the top 10 productive countries (Figures 2B,C). Moreover, the lack of Chinese institutes and scholars in the top 10 productive institutes and authors can be observed (Figure 3), and China is a late starter in international cooperation (Figure 2E). These results indicate less influence of Chinese scholars on the uremic cardiomyopathy field, and it is manifestly unreasonable due to the existence of the largest population with CKD in China (35). In China, most nephrologists concentrate on glomerulonephritis, acute kidney injury, CKD, and dialysis (18, 36), while the investigations on uremic cardiomyopathy started fairly late, as indicated by the most influential study on uremic cardiomyopathy published by Jinghong Zhao’s group at 2015 (cited 105) (37). Therefore, Chinese scholars exhibit less influence in this field, and they should be more involved in uremic cardiomyopathy research with the aid of ample clinical resources.

### Co-authorship Analysis in Uremic Cardiomyopathy Research

Myles Wolf of the University of Miami plays a leading role in the investigations of the relationship between phosphate metabolism and uremic cardiomyopathy, and he is a pioneer of the most influential breakthrough in uremic cardiomyopathy (FGF-23 and uremic cardiomyopathy), as indicated by two studies from his group (27, 38). Based on this achievement, Myles Wolf established the largest co-authorship network in uremic cardiomyopathy (Figure 3D). Joseph I. Shapiro of Marshall University mainly concentrates on cardiotonic steroids and uremic cardiomyopathy; his group found that CKD-induced cardiotonic steroids elevation is sufficient to induce LVH and cardiac fibrosis through activating redox-sensitive Na^+^-K^+^-ATPase (39). However, although Joseph I. Shapiro published lots of studies on uremic cardiomyopathy, his group has failed to possess a central position in uremic cardiomyopathy (Figure 3D). This also influences the cooperation between Joseph I. Shapiro’s group and the outside group (Figure 3D). Notably, these two scholars both came from institutions in the United States, indicating the leading role of the United States in uremic cardiomyopathy research on the secondary side.

### The Hint From the Distribution of Journals

Based on the number, TLCS, TGCS, and H index, the Nephrology Dialysis Transplantation, American Journal of Kidney Diseases, Kidney International, and the Journal of the American Society of Nephrology are the most influential journals for scholars in the uremic cardiomyopathy field (Table 4). These results also imply that the journals in the nephrology domain, rather than journals in the cardiology domain, possess a central role in publishing uremic cardiomyopathy studies. Interestingly, we can also find highly influential publications in non-nephrology journals *via* citation burst analysis, including New England Journal of Medicine, Journal of the American Medical Association, Annals of Internal Medicine, Journal of Clinical Investigation, and Cell Metabolism (Figure 5B), indicating that interdisciplinary journals also publish breakthrough articles on uremic cardiomyopathy. This may be attributed to the interdisciplinary property of uremic cardiomyopathy, which attracts the attention of both cardiologists and nephrologists. Hence, it suggests that interdisciplinary collaboration is required for further investigations as well.

### The Variation Tendency Concerned Topic in the Uremic Cardiomyopathy Field

Uremic cardiomyopathy is a concept with a long history, and it can be dated back to the age of Richard Bright, who firstly reported CKD and observed pericarditis and cardiac hypertrophy in patients with CKD (40). Although the feature of uremic cardiomyopathy is observed earlier, the impact of this change on patients with CKD is unclear until the 1990s. In 1995, Foley et al. reported that LVH can affect mortality in patients with end-stage renal disease (2, 3), and this leads to a rapidly increasing trend of publications in the uremic cardiomyopathy field. Subsequently, anemia is found to be related to uremic cardiomyopathy (25), and the protective effect of erythropoietin makes it the earliest therapeutic strategy for uremic cardiomyopathy (9) (Figure 5A). However, erythropoietin treatment is recognized to cause hypertension in CKD (41), thus, it is not an ideal drug for uremic cardiomyopathy. Consistent with this, the analysis from Lv et al. (34) demonstrated that anemia is an early topic in cardiorenal syndrome research further implies anemia is an early recognized factor affecting both the kidney and the heart. Vitamin D supplementation moves into center stage of uremic cardiomyopathy due to its deficiency is linked to cardiovascular events (Figure 5A) (42). Unfortunately, Thadhani et al. demonstrated that 48-week vitamin D therapy is unable to mitigate LVH and improve diastolic dysfunction in patients with CKD (43); thus, the attraction of vitamin D drops rapidly around 2013 (Figure 5A).

**FIGURE 7:**
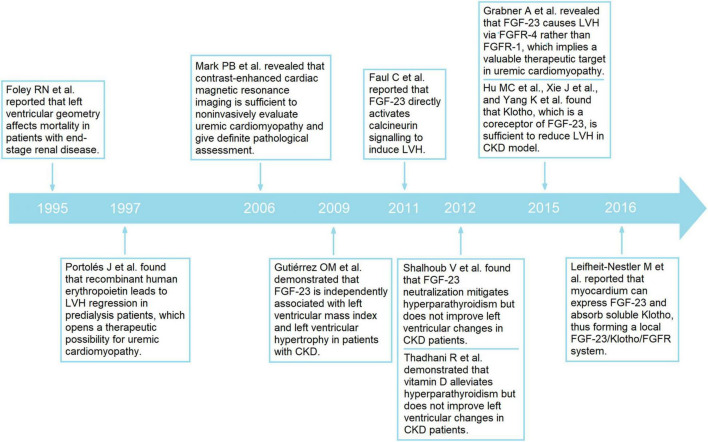
Major achievements in the uremic cardiomyopathy field.

Importantly, Gutiérrez et al. (27) reported that FGF-23 is independently associated with LVH in patients with CKD, and Faul et al. (12) further found that FGF-23 activates calcineurin signaling to induce LVH (Figure 5A). These results bring another therapeutic strategy for uremic cardiomyopathy; however, neutralizing FGF-23 improves hyperparathyroidism yet increases mortality, and the latter is caused by more severe hyperphosphatemia and vascular calcification (14). This temporarily delays the progress in FGF-23 and uremic cardiomyopathy. Fortunately, Grabner et al. (24) found that FGF-23 induces LVH *via* FGFR-4 instead of FGFR-1, which is critical for the phosphate metabolic effects of FGF-23, hence indicating that FGFR-4 inhibitor can be used to treat uremic cardiomyopathy. Strangely, there are no clinical trials based on FGFR-4 inhibitors have been conducted now. Most recent progress on uremic cardiomyopathy is mainly based on studies performed by Hu et al. (29) and Leifheit-Nestler et al. (30), who found that soluble klotho can alleviate LVH in CKD. Further investigations found the protective effects of soluble klotho are FGF-23-dependent and FGF-23-independent (28), thus, implying another therapeutic strategy for uremic cardiomyopathy (Figures 5A, 7).

Notably, the timeline view of references also showed that investigations related to uremic cardiomyopathy concentrate on both basic and clinical studies (Figure 5A), and this is confirmed by burst detection, which showed that most influential publications are presented in both basic and clinical journals (Figure 5B). Indeed, the investigations on uremic cardiomyopathy exhibit a good connection between basic and clinical studies, as indicated by a relatively rapid translation of basic to the clinic, and this brings a favorable developmental pattern to the uremic cardiomyopathy field.

### Outlook of Uremic Cardiomyopathy

Phosphate metabolism disorder exerts a central role in uremic cardiomyopathy, and it is now considered as a core to treat uremic cardiomyopathy (44). Relevant to this, studies on FGF-23 and its coreceptor klotho, which are closely associated with phosphate metabolism disorder, accounted for 40% (4/10) of publications with top 10 TLCS (Table 5). Lowering phosphate levels through dietary phosphate restriction, dialysis, and phosphate binders may mitigate uremic cardiomyopathy. Although phosphate binders are the only pharmacological treatment for hyperphosphatemia until now, Chue et al. (45) demonstrated that 40-week treatment wih phosphate binder, sevelamer, is unable to alleviate arterial stiffness, left ventricular mass, or any parameters of left ventricular systolic and diastolic function in patients with stage 3 CKD. In addition, hyperphosphatemia-induced FGF-23 elevation and klotho deficiency can also be therapeutic targets for uremic cardiomyopathy. Nevertheless, the complicated relationship between FGF-23/klotho and uremic cardiomyopathy hampers related progress (28). Soluble klotho supplementation and FGFR-4 blockade may act as therapeutic strategies as well (24, 29, 30). Hence, FGF-23 and klotho still receive wide attention in recent years (Figures 5A, 6A,C), and phosphate metabolism disorder will remain the hotspot of uremic cardiomyopathy in the coming time. Consistently, FGF-23 and klotho are the key nodes in Lv et al. (34) keyword occurrence analysis, and they also thought these should be important in future cardiorenal syndrome research.

Although phosphate metabolism disorder possesses the center stage of uremic cardiomyopathy, some potential aspects may progress this field as well. CKD is often accompanied by sodium retention, which causes a class of endogenous natriuretic factors, named endogenous cardiotonic steroids (eCTS), to promote renal sodium excretion (46). The eCTS can bound to Na^+^-K^+^-ATPase but does not affect its ion transport capacity (47), whereas, eCTS activates Src kinase, which is interacted with Na^+^-K^+^-ATPase, to activate prohypertrophic signaling activation and ROS generation, thus, promoting cardiac hypertrophy and fibrosis (48, 49). Based on these, targeting Na^+^-K^+^-ATPase or even eCTS to alleviate uremic cardiomyopathy may be effective.

Identifying novel biomarkers or risk factors of uremic cardiomyopathy is urgent in clinical practice. Serum troponin T and nocturnal hypertension may be novel indicators associated with heart failure in patients. Recently, Janus et al. (50) reported that a panel of brain natriuretic peptides, FGF-23, fibrinogen, and high-sensitive troponin T can be used to predict heart failure in patients with CKD. In addition, Fu et al. (51) found that nocturnal hypertension is associated with LVH in patients with CKD, indicating that ambulatory blood pressure monitoring may be used as an important indicator to evaluate the risk of uremic cardiomyopathy. Further studies are required to confirm these.

### Limitations

Our research has several limitations. Uremic cardiomyopathy is a clinical diagnosis with ambiguous diagnostic criteria. Although we enriched the search strategy as much as possible, such as adding “cardiac hypertrophy” and “left ventricular hypertrophy” as search terms. Still, fewer studies about uremic cardiomyopathy were not included in the analysis, such as case reports, which may give different results but are less included in SCIE. Moreover, most results were based on machine algorithms, which may have slight deviations. Finally, future analysis was based on the co-occurrence of keywords, whereas some potential aspects may temporarily not co-occur with uremic cardiomyopathy.

## Data Availability Statement

The original contributions presented in the study are included in this article/Supplementary Material, further inquiries can be directed to the corresponding author.

## Ethics statement

Ethical review and approval was not required for this study in accordance with the local legislation and institutional requirements.

## Author Contributions

J-FB and AL designed the investigation. J-FB, Q-YS, and DZ collected the data. J-FB, P-PH, and J-JM analyzed the data. J-FB drafted the manuscript. AL revised the manuscript. All authors contributed to the article and approved the final version of the manuscript.

## Conflict of Interest

The authors declare that the research was conducted in the absence of any commercial or financial relationships that could be construed as a potential conflict of interest.

## Publisher’s Note

All claims expressed in this article are solely those of the authors and do not necessarily represent those of their affiliated organizations, or those of the publisher, the editors and the reviewers. Any product that may be evaluated in this article, or claim that may be made by its manufacturer, is not guaranteed or endorsed by the publisher.
