# Supplementary material for: A Bibliometric and Visualized Analysis of Uremic Cardiomyopathy From 1990 to 2021
Source: Front Cardiovasc Med. 2022 Jul 12;9:908040. doi: 10.3389/fcvm.2022.908040 (PMC9314665; doi:10.3389/fcvm.2022.908040)
Supplement: Supplementary file 1 [file Table_1.DOCX]

Supplementary Table 1. Publication types

| Document Type | Number | TLCS | TGCS |
| --- | --- | --- | --- |
| Article | 1810 | 8602 | 72064 |
| Review | 593 | 1652 | 36446 |

Supplementary Table 2. The top 10 publications with highest TGCS

| Rank | First author | Journal | Year | Category | TLCS | TGCS |
| --- | --- | --- | --- | --- | --- | --- |
| 1 | Giuseppe Mancia | Journal of Hypertension | 2013 | Guideline | 35 | 3545 |
| 2 | Robert N Foley | American Journal of Kidney Diseases | 1998 | Epidemiology | 190 | 2631 |
| 3 | Giuseppe Mancia | European Heart Journal | 2013 | Guideline | 19 | 2396 |
| 4 | Bryan Williams | European Heart Journal | 2018 | Guideline | 9 | 2049 |
| 5 | Paul K Whelton | Hypertension | 2018 | Guideline | 7 | 1738 |
| 6 | Markus Wyss | Physiol Rev | 2000 | Review | 0 | 1688 |
| 7 | Christian Faul | Journal of Clinical Investigation | 2011 | Epidemiology/Pathophysiology | 373 | 1320 |
| 8 | Claudio Ronco | Journal of the American College of Cardiology | 2008 | Review-General view | 54 | 1261 |
| 9 | Bryan Williams | Journal of Hypertension | 2018 | Guideline | 8 | 1096 |
| 10 | Ron T Gansevoort | Lancet | 2013 | Review- Epidemiology | 43 | 1089 |
